# Spin Exchanges between Transition Metal Ions Governed by the Ligand p-Orbitals in Their Magnetic Orbitals

**DOI:** 10.3390/molecules26030531

**Published:** 2021-01-20

**Authors:** Myung-Hwan Whangbo, Hyun-Joo Koo, Reinhard K. Kremer

**Affiliations:** 1 Department of Chemistry and Research Institute for Basic Sciences, Kyung Hee University, Seoul 02447, Korea; hjkoo@khu.ac.kr; 2 Department of Chemistry, North Carolina State University, Raleigh, NC 27695-8204, USA; 3 Max Planck Institute for Solid State Research, Heisenbergstrasse 1, D-70569 Stuttgart, Germany; rekre@fkf.mpg.de

**Keywords:** **Keywords**: spin exchange, magnetic orbitals, ligand p-orbital tails, M–L–M exchange, M–L…L–M exchange, α-CuV_2_O_6_, LiCuVO_4_, (CuCl)LaNb_2_O_7_, Cu_3_(CO_3_)_2_(OH)_2_

## Abstract

In this review on spin exchanges, written to provide guidelines useful for finding the spin lattice relevant for any given magnetic solid, we discuss how the values of spin exchanges in transition metal magnetic compounds are quantitatively determined from electronic structure calculations, which electronic factors control whether a spin exchange is antiferromagnetic or ferromagnetic, and how these factors are related to the geometrical parameters of the spin exchange path. In an extended solid containing transition metal magnetic ions, each metal ion M is surrounded with main-group ligands L to form an ML_n_ polyhedron (typically, n = 3–6), and the unpaired spins of M are represented by the singly-occupied d-states (i.e., the magnetic orbitals) of ML_n_. Each magnetic orbital has the metal d-orbital combined out-of-phase with the ligand p-orbitals; therefore, the spin exchanges between adjacent metal ions M lead not only to the M–L–M-type exchanges, but also to the M–L…L–M-type exchanges in which the two metal ions do not share a common ligand. The latter can be further modified by d^0^ cations A such as V^5+^ and W^6+^ to bridge the L…L contact generating M–L…A…L–M-type exchanges. We describe several qualitative rules for predicting whether the M–L…L–M and M–L…A…L–M-type exchanges are antiferromagnetic or ferromagnetic by analyzing how the ligand p-orbitals in their magnetic orbitals (the ligand p-orbital tails, for short) are arranged in the exchange paths. Finally, we illustrate how these rules work by analyzing the crystal structures and magnetic properties of four cuprates of current interest: α-CuV_2_O_6_, LiCuVO_4_, (CuCl)LaNb_2_O_7_, and Cu_3_(CO_3_)_2_(OH)_2_.

## 1. Introduction

**Figure 1:**
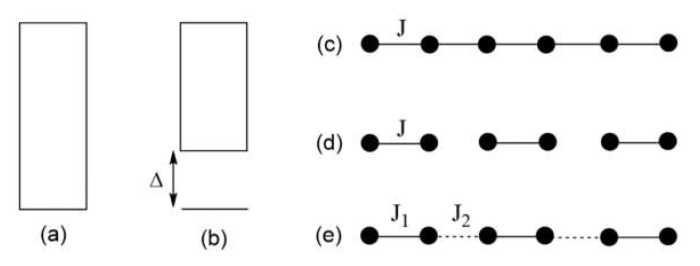
(**a**,**b**) Allowed energy states of a magnetic solid. Between the lowest-lying excited state and the ground state, there is no energy gap in (**a**), but a non-zero energy gap in (**b**). (**c**–**e**) Examples of simple spin lattices: a uniform chain in (**c**); isolated spin dimers in (**d**); and an alternating chain in (**e**). Here, all nearest-neighbor spins are antiferromagnetically coupled.

An extended solid consisting of transition metal magnetic ions has closely packed energy states (Figure 1a,b) so that, at a given non-zero temperature, the ground state as well as a vast number of the excited states can be thermally occupied. The thermodynamic properties such as the magnetic susceptibility and the specific heat of a magnetic system represents the weighted average of the properties associated with all thermally occupied states, with their Boltzmann factors as the weights. Such a quantity is difficult to calculate if all states were to be determined by first principle electronic structure calculations. 

To generate the states of a given magnetic system and subsequently calculate the thermally-averaged physical property, a model Hamiltonian (also called a toy Hamiltonian) is invariably employed [1,2,3]. A typical model Hamiltonian used for this purpose is the Heisenberg-type spin Hamiltonian, H_spin_, expressed as:

(1) $$H_{spin}=\sum_{i>j}J_{ij}{\hat{S}}_{i}\cdot {\hat{S}}_{j}$$

where the energy spectrum of a magnetic system as the sum of the pairwise spin exchange interactions $J_{ij}{\hat{S}}_{i}\cdot {\hat{S}}_{j}$ is approximated. The spin operators ${\hat{S}}_{i}\;\mathrm{and}\;{\hat{S}}_{j}$ (at the spin sites i and j, respectively) can be treated as the spin vectors ${\vec{S}}_{i}\;\mathrm{and}\;{\vec{S}}_{j}$, respectively, unless they operate on spin states. If all magnetic ions of a given system are identical with spin S, each term ${\vec{S}}_{i}\cdot {\vec{S}}_{j}$ can be written as ${\vec{S}}_{i}\cdot {\vec{S}}_{j}=S^{2}\cos\theta_{ij}$ where $\theta_{ij}$ is the angle between the two spin vectors. In such a case, Equation (1) is rewritten as:

(2) $$H_{spin}=\sum_{i>j}J_{ij}S^{2}cos\theta_{ij}$$

In a collinearly ordered spin arrangement of a magnetic solid, every two spin arrangements are either ferromagnetic (FM, i.e., parallel ($\theta_{ij}=0{}^{\circ}$)) or antiferromagnetic (AFM, i.e., antiparallel ($\theta_{ij}=180{}^{\circ}$)). With the definition of a spin Hamiltonian as in Equation (1), AFM and FM spin exchanges are represented by positive and negative J_ij_ values, respectively. For any collinearly ordered spin arrangement, the total energy is readily written as a function of the various spin exchanges J_ij_.

The energy spectrum allowed for a magnetic system, and hence its magnetic properties, depend on its spin lattice. The latter refers to the repeat pattern of predominant spin exchange paths, i.e., those with large |J_ij_| values. For example, between the ground and the excited states, a uniform half-integer spin AFM chain (Figure 1c) has no energy gap (Figure 1a), whereas an isolated AFM dimer (Figure 1d) and an alternating AFM chain (Figure 1e) have a non-zero energy gap (Figure 1b). The spin lattice of a given magnetic solid is determined by its electronic structure, which makes it interesting how to identify the spin lattice of a magnetic solid on the basis of its atomic and electronic structures. Strong AFM exchanges between magnetic ions are often termed magnetic bonds, in contrast to chemical bonds determined by strong chemical bonding. Thus, Figure 1c represents a uniform AFM chain, and Figure 1d represents isolated AFM dimers. The magnetic bonds do not necessarily follow the geometrical pattern of the magnetic ion arrangement dictated by chemical bonding.

**Figure 2:**
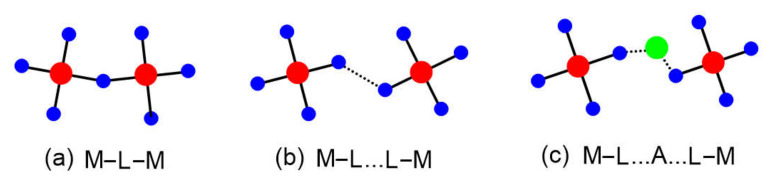
Three types of spin exchange paths associated with two magnetic ions: (**a**) M–L–M, (**b**) M–L…L–M, and (**c**) M–L…A…L–M, where A represents a d^0^ cation such as V^5+^ or W^6+^. The M, L, and A are represented by red, blue, and green circles, respectively.

In a magnetic solid, transition metal ions M often share a common ligand L to form M–L–M bridges (Figure 2a). The spin exchange between the magnetic ions in an M–L–M bridge has been termed “superexchange” [4,5,6,7]. Whether the spin arrangement between two metal ions becomes FM or AFM, as described by the Goodenough–Kanamori rules formulated in the late 1950s, depends on the geometry of the M–L–M bridge [5,6,7,8,9]. Ever since, the Goodenough–Kanamori rules have greatly influenced the thinking of inorganic and solid-state chemists dealing with magnetic systems. Since the early 2000s, it has become increasingly clear that the magnetic properties of certain compounds cannot be conclusively explained unless also one takes into consideration the spin exchanges of the M–L…L–M (Figure 2b) or the M–L…A…L–M (Figure 2c) types, termed super-superexchanges [1,2,3], in which the metal ions do not share a common ligand. In the late 1950s, it was impossible to imagine that spin exchanges could take place in such paths in which the M…M distances were very long, because the prevailing concept of chemical bonding at that time, mainly based on the valence bond picture [10], suggested that an unpaired electron of a magnetic ion is accommodated in a pure d-orbital of M. In an extended solid, each transition metal ion M is typically surrounded with main-group ligand atoms L to form an ML_n_ polyhedron (n = 3–6), and each unpaired electron of ML_n_ resides in a singly-occupied d-state (referred to as a magnetic orbital) of ML_n_, in which the d-orbital of M is combined out-of-phase with the p-orbitals of L. In this molecular orbital picture, the unpaired spin density is already delocalized from the d-orbital of M (the “magnetic orbital head”) to the p-orbitals of L (the “magnetic orbital tails”) [1,2,3]. Thus, it is quite natural to think that the spin exchange between the metal ions in an M–L…L–M path takes place through the overlap between the ligand p-orbital tails present in the L…L contact. This interaction between the p-orbital tails can be modified by the empty d_π_ orbitals of the d^0^ cation A (e.g., V^5+^ and W^6+^) in an M–L…A…L–M exchange.

Given that spin exchanges between magnetic ions are determined by the p-orbital tails of their magnetic orbitals, it is not surprising that spin exchanges of the M–L…L–M or M–L…A…L–M type can be stronger than those of the M–L–M type, and that spin exchanges are generally strong only along a certain direction of the crystal structure. Although this aspect has been repeatedly pointed out in review articles over the years [1,2,3], it is still not infrequent to observe that experimental results are incorrectly interpreted simply because spin lattices have been deduced considering only the M–L–M type spin exchanges. Such an unfortunate mishap is akin to providing solutions in search of a problem. In this review, written as a tribute to John B. Goodenough for his long and illustrious scientific career culminating with the Nobel Prize in 2019, we review what electronic factors govern the nature of the M–L–M, M–L…L–M and M–L…A…L–M type spin exchanges, with an ultimate goal to provide several qualitative rules useful for finding the spin lattice relevant for any given magnetic system.

Our work is organized as follows: Section 2 examines how to quantitatively determine the values of spin exchanges by carrying out the energy-mapping analysis based on electronic structure calculations. Section 3 explores the electronic factors controlling whether a spin exchange is AFM or FM. In Section 4, we derive several qualitative rules that enable one to predict whether the M–L…L–M and M–L…A…L–M type spin exchanges are AFM or FM by analyzing how their ligand p-orbitals are arranged in the exchange paths. To illustrate how the resulting structure–property relationships operate, in Section 5 we examine the crystal structures and magnetic properties of α-CuV_2_O_6_, LiCuVO_4_, (CuCl)LaNb_2_O_7_ and Cu_3_(CO_3_)_2_(OH)_2_. Our concluding remarks are presented in Section 6.

## 2. Energy Mapping Analysis for Quantitative Evaluation of Spin Exchanges

This section probes how to define the relevant spin Hamiltonian for a given magnetic system on a quantitative level. This requires the determination of the spin exchanges to include in the spin Hamiltonian. For various collinearly ordered spin states of a given magnetic system, one finds the expressions for their relative energies in terms of the spin exchange parameters J_ij_ to evaluate, performs DFT+U [11] or DFT+hybrid [12] electronic structure calculations for the ordered spin states to determine the numerical values for their relative energies, and finally maps the two sets of relative energies to find the numerical values of the exchanges.

### 2.1. Using Eigenstates

**Figure 3:**
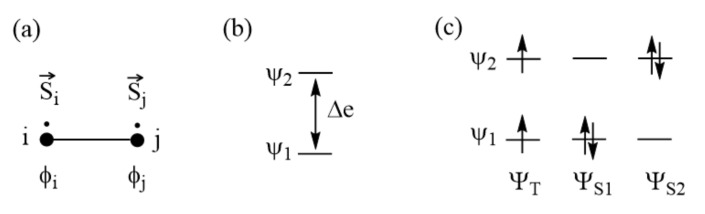
(**a**) Spin dimer made up of two S = 1/2 ions at sites i and j. Each site has one unpaired spin. The magnetic orbitals at the sites i and j are represented by ϕ_i_ and ϕ_j_, respectively. (**b**) The bonding and antibonding states, Ψ_1_ and Ψ_2_, resulting from the interactions between ϕ_i_ and ϕ_j_, are split in energy by Δe. (**c**) The singlet and triplet electron configurations resulting from Ψ_1_ and Ψ_2_.

To gain insight into the meaning and the nature of a spin exchange, we examine a spin dimer made up of two S = 1/2 ions (Figure 3a). The spin Hamiltonian describing the energies of this dimer is given by:

(3) $$H_{spin}=J{\hat{S}}_{i}\cdot {\hat{S}}_{j},$$

The spin states allowed for this dimer are the singlet and triplet states, $\Psi_{S}$ and $\Psi_{T}$, respectively.

(4) $$\begin{array}{l} \Psi_{T}=|↑↑\rangle ,\;|↓↓\rangle ,\;\left(|↑↓\rangle +|↓↑\rangle \right)/\sqrt{2}\\ \Psi_{S}=\;\left(|↑↓\rangle -|↓↑\rangle \right)/\sqrt{2} \end{array}$$

It can be readily shown that these states are the eigenstates of the spin Hamiltonian by rewriting Equation (3) as:

(5) $$H_{spin}=J{\hat{S}}_{iz}{\hat{S}}_{jz}+(J/2)[{\hat{S}}_{i+}{\hat{S}}_{j-}+{\hat{S}}_{i-}{\hat{S}}_{j+}]$$

where ${\hat{S}}_{mz}$ (m = i, j) is the z-component of ${\hat{S}}_{m}$, while ${\hat{S}}_{m+}$ and ${\hat{S}}_{m-}$ are the raising and lowering operators associated with ${\hat{S}}_{m}$, respectively. Then, it is found that:

(6) $$\begin{array}{l} H_{spin}\Psi_{T}=(J/4)\Psi_{T}\\ H_{spin}\Psi_{S}=(-3J/4)\Psi_{S} \end{array}$$

Thus:

(7) $$\Delta E_{eigen}=E_{T}-E_{S}=\langle \Psi_{T}|H_{spin}|\Psi_{T}\rangle -\langle \Psi_{S}|H_{spin}|\Psi_{S}\rangle =J$$

**Figure 4:**
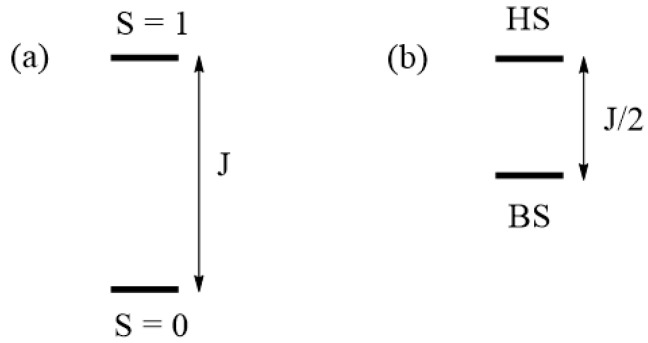
Relationships of the spin exchange J to the energy difference between two spin states of a spin dimer made up of two S = 1/2 ions in terms of (**a**) the eigenstates and (**b**) the broken-symmetry states. The legends HS and BS in (**b**) refer to the high-symmetry and broken-symmetry states, respectively.

Therefore, the spin exchange J is related to the energy difference between the singlet and triplet states of the spin dimer, as illustrated in Figure 4a.

### 2.2. Using Broken-Symmetry States

In general, it is not an easy task to find the eigenstates of a general spin Hamiltonian (e.g., Equation (1)), which makes it difficult to relate the spin exchanges to the energy differences between the eigenstates of a magnetic system. However, the energies for the broken-symmetry (BS) states (i.e., the non-eigenstates) of a spin Hamiltonian are easy to evaluate. For the magnetic dimer in Section 2.1, the high-spin (HS) state, $\Psi_{HS}=|↑↑\rangle \;\mathrm{or}\;|↓↓\rangle$, which has an FM spin arrangement, is an eigenstate of the spin Hamiltonian (Equation (3)). However, the low-spin (LS) state can be expressed as:

$$\Psi_{LS}=\;|↑↓\rangle \;\mathrm{or}\;|↓↑\rangle$$

This state has an AFM spin arrangement and is the BS state of the spin Hamiltonian. Using Equation (5), it is found that:

(8) $$\begin{array}{l} H_{spin}|↑↓\rangle =(-J/4)\;|↑↓\rangle +(J/2)\;|↓↑\rangle \\ H_{spin}|↓↑\rangle =(-J/4)\;|↓↑\rangle +(J/2)\;|↑↓\rangle \end{array}$$

Therefore:

(9) $$\langle ↑↓|H_{spin}|↑↓\rangle =\langle ↓↑|H_{spin}|↓↑\rangle =-J/4$$

From Equations (6) and (9), we obtain:

(10) $$\Delta E_{BS}=\langle \Psi_{HS}|H_{spin}|\Psi_{HS}\rangle -\langle \Psi_{BS}|H_{spin}|\Psi_{BS}\rangle =J/2$$

The spin exchange J is related to the energy difference between the states including the BS states (Figure 4b).

### 2.3. Energy Mapping

For a general spin lattice, the energy difference between any two states (involving BS and HS states) can be readily determined by using the spin Hamiltonian of Equation (3), which is expressed as a function of unknown constants J_ij_. To evaluate the spin exchanges J_ij_ of a general spin Hamiltonian, it is necessary to numerically determine the relative energies of various ordered spin states. This is done by performing DFT+U [11] or DFT+hybrid [12] electronic structure calculations for the collinearly ordered spin states of a magnetic system on the basis of the electronic Hamiltonian, H_elec_. These types of calculations ensure that the electronic structures calculated for various ordered spin states have a bandgap as expected for a magnetic insulator. Suppose that N different spin exchange paths J_ij_ are considered to describe a given magnetic solid. If one considers N + 1 ordered spin states, for example, one can determine N different relative energies ΔE_spin_(i) (i = 1, 2,⋅⋅⋅, N) expressed in terms of N different spin exchanges J_ij_ (in principle, one could use more spin states, but at least N+1 are necessary; using more would produce error bars and increase the precision of the analysis). By carrying out electronic structure calculations for the N+1 ordered spin states of the magnetic system, one obtains the numerical values for the N different relative energies ΔE_elec_(i) (i = 1, 2,⋅⋅⋅, N). Then, by equating the ΔE_spin_(i) (i = 1, 2, ⋅⋅⋅, N) values to the corresponding ΔE_elec_(i) (i = 1, 2, ⋅⋅⋅, N) values, the N different spin exchanges J_ij_ are obtained.

ΔE_spin_(i) (i = 1, 2, ⋅⋅⋅, N) ↔ ΔE_elec_(i) (i = 1, 2, ⋅⋅⋅, N) (11)

The spin exchanges discussed so far are known as Heisenberg exchanges. There are other variants of interactions between spins which, though weaker than Heisenberg exchanges in strength, are needed to explain certain magnetic properties not covered by the symmetrical Heisenberg exchanges. They include Dzyaloshinskii–Moriya exchanges (or antisymmetric exchanges) and asymmetric exchanges [2,3]. The energy-mapping analysis based on collinearly ordered spin states allows one to determine the Heisenberg spin exchanges only. To evaluate the Dzyaloshinskii–Moriya and asymmetric spin exchanges, the energy-mapping analysis employs the four-state method [2,13], in which non-collinearly ordered broken-symmetry states are used. A further generalization of this energy-mapping method was developed to enable the evaluation of other energy terms that one might include in a model spin Hamiltonian [14].

## 3. Qualitative Features of Spin Exchange

A spin Hamiltonian appropriate for a given magnetic system is one that consists of the predominant spin exchange paths. Such a spin Hamiltonian can be determined by evaluating the values of various possible spin exchanges for the magnetic system by performing the energy-mapping analysis as described in Section 2. If the spin lattice is chosen without quantitatively evaluating its spin exchanges, one might inadvertently choose a spin lattice irrelevant for the interpretation of the experimental data. When simulating the thermodynamic properties using a chosen set of spin exchanges, the values of the spin exchanges are optimized until they provide the best possible simulation even if the chosen spin lattice is incorrect from the viewpoint of electronic structure. Thus, in principle, more than one spin lattice might provide an equally good simulation. In interpreting the experimental results of a magnetic system with a correct spin lattice, it is crucial to know what electronic and structural factors control the signs and the magnitudes of spin exchanges.

### 3.1. Parameters Affecting Spin Exchanges

To examine what energy parameters govern the sign and magnitude of a spin exchange, we revisit the spin exchange of the spin dimer (Figure 3a) by explicitly considering the electronic structures of its singlet and triplet states. For simplicity, we represent each spin site with one magnetic orbital. As will be discussed in Section 5, the nature of the magnetic orbital plays a crucial role in determining the sign and magnitude of a spin exchange. We label the magnetic orbitals located at the spin sites i and j as ϕ_i_ and ϕ_j_, respectively (Figure 3a). These orbitals overlap weakly, and hence interact weakly, to form the in-phase and out-of-phase states, $\Psi_{1}$ and $\Psi_{2}$, respectively, with energy split Δe between the two (Figure 3b). The overlap integral $\langle \phi_{i}|\phi_{j}\rangle$ between ϕ_i_ and ϕ_j_ is small for magnetic systems, so $\Psi_{1}$ and $\Psi_{2}$ are well approximated by:

(12) $$\Psi_{1}\;\approx \frac{\left(\phi_{i}+\phi_{j}\right)}{\sqrt{2}},\;\Psi_{2}\;\approx \;\frac{\left(\phi_{i}-\phi_{j}\right)}{\sqrt{2}}$$

For simplicity, it is assumed here that the in-phase combination (i.e., the bonding combination) is described by the plus combination, which amounts to the assumption that the overlap integral $\langle \phi_{i}|\phi_{j}\rangle$ is positive. The energy split Δe is approximately proportional to the overlap integral:

(13) $$\Delta e\propto \langle \phi_{i}|\phi_{j}\rangle$$

In understanding the qualitative features describing the electronic energy difference between the singlet and triplet states, ΔE_elec_, and hence the spin exchange J, it is necessary to consider two other quantities. One is the on-site repulsion U_ii_:

(14) $$U_{ii}=\langle \phi_{i}(1)\phi_{i}(2)\left|1/r_{12}\right|\phi_{i}(1)\phi_{i}(2)\rangle \;(i\;=1,\;2)$$

where the product $\phi_{i}(m)\phi_{i}(m)$ is the electron density $\rho_{ii}(m)$ associated with the orbital $\phi_{i}(m)$ occupied by electron m (=1, 2). Thus, U_ii_ is the self-repulsion when the orbital $\phi_{i}$ is occupied by two electrons. If the spin sites i and j are identical in nature, the on-site repulsion U_jj_ at the site j is the same as U_ii_, therefore it is convenient to use the symbol U to represent both U_ii_ and U_jj_ (i.e., U = U_ii_ = U_jj_). The other quantity of interest is the exchange repulsion K_ij_ between ϕ_i_ and ϕ_j_:

(15) $$K_{\mathrm{ij}}=\;\langle \phi_{i}(1)\phi_{j}(2)\left|1/r_{12}\right|\phi_{j}(1)\phi_{i}(2)\rangle$$

This is the self-repulsion arising from the overlap electron density:

(16) $$\rho_{\mathrm{ij}}(m)\;=\phi_{i}(m)\phi_{j}(m)\;(m\;=\;1,\;2)$$

**Figure 5:**
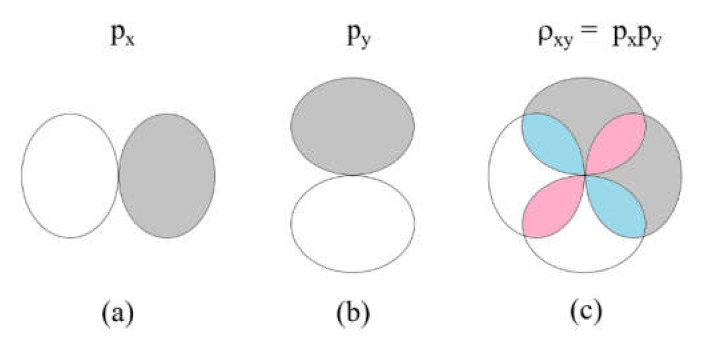
The overlap density resulting from two p-orbital at a given atomic site: (**a**) a p_x_ orbital; (**b**) a p_y_ orbital; and (**c**) the overlap density between the two orbitals, $\rho_{xy}=p_{x}p_{y}$. The pink and cyan regions have positive and negative values, respectively.

To illustrate the difference between the overlap integral and overlap electron density, we consider the $p_{x}$ and $p_{y}$ atomic orbitals located at a same atomic site (Figure 5a,b). The product $p_{x}p_{y}$ represents the overlap electron density $\rho_{xy}$, which consists of four overlapping regions (Figure 5c); two regions of positive electron density (colored in pink) and two regions of negative electron density (colored in cyan). The overlap integral $\langle p_{x}|p_{y}\rangle$ is the sum of these four overlap electron densities, which adds up to zero.

The exchange repulsion between $p_{x}$ and $p_{y}$ is written as:

(17) $$K_{xy}=\langle p_{x}(1)p_{x}(2)\left|1/r_{12}\right|p_{y}(1)p_{y}(2)\rangle$$

Equation (17) is given by the sum of the self-repulsion resulting from each overlapping region of the overlap electron density $\rho_{xy}$ (Figure 5c). Each overlapping region, be it positive or negative, leads to a positive repulsion; therefore, the exchange repulsion $K_{xy}$ is positive.

### 3.2. Two Competing Components of Spin Exchange

In Figure 3c, the configuration $\Psi_{T}$ represents the triplet state of the dimer while the configurations $\Psi_{S1}$ and $\Psi_{S2}$ each represent a singlet state. For a magnetic system, for which Δe is very small, the singlet state is not well described by $\Psi_{S1}$ alone and is represented by a linear combination of $\Psi_{S1}$ and $\Psi_{S2}$, i.e., $\Psi_{S}=c_{1}\Psi_{S1}+c_{2}\Psi_{S2}$, where the mixing coefficients c_1_ and c_2_ are determined by the interaction between $\Psi_{S1}$ and $\Psi_{S2}$, namely, $\langle \Psi_{S1}|H_{elec}|\Psi_{S2}\rangle =K_{ij}$, as well as by the energies of $\Psi_{S1}$ and $\Psi_{S2}$, that is, $\langle \Psi_{S1}|H_{elec}|\Psi_{S1}\rangle =E_{1}\;$ and $\langle \Psi_{S2}|H_{elec}|\Psi_{S2}\rangle =E_{2}$. After some lengthy manipulations under the condition, K_ij_ >> |E_1_−E_2_|, which is satisfied for magnetic systems, the energy difference between the triplet and singlet states, and hence the spin exchange J, is expressed as [15]:

(18) $$J=E_{T}-E_{S}\approx -2K_{ij}+\frac{{\left(\Delta e\right)}^{2}}{U}$$

Note that the spin exchange J consists of two components, $J=J_{F}+J_{AF}$, where:

(19) $$\begin{array}{l} J_{F}=-2K_{ij}<0\\ J_{AF}=\frac{{\left(\Delta e\right)}^{2}}{U}>0 \end{array}$$

The magnitude of the FM component | $J_{F}$| increases as the exchange repulsion $K_{ij}$ increases, namely, as the overlap density $\left|\rho_{ij}\right|=\left|\phi_{i}\phi_{j}\right|$ increases. The strength of the AFM component $J_{AF}$ increases with increasing the energy split Δe, i.e., with increasing the overlap integral $\langle \phi_{i}|\phi_{j}\rangle$, while $J_{AF}$ decreases as the on-site repulsion U increases.

As already discussed in Section 2, the quantitative values of spin exchanges can be accurately determined by the energy-mapping analysis based on first-principles DFT+U or DFT+hybrid calculations. The purpose of Equations (18) and (19) is not to determine the numerical value of any spin exchange, but to show that each spin exchange J consists of two competing components, J_F_ and J_AF_, that the overall sign of J is determined by which component dominates, and which electronic parameters govern the strength of each component.

## 4. Spin Exchanges Determined by the Ligand p-Orbitals in the Magnetic Orbitals

**Figure 6:**
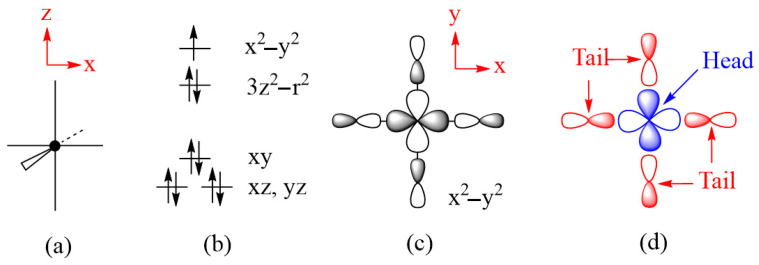
(**a**) An axially elongated CuL_6_ octahedron. (**b**) The electron configuration of a Cu^2+^(d^9^) ion at an axially elongated octahedral site. (**c**) The magnetic orbital of the Cu^2+^(d^9^) ion at an axially elongated octahedral site, which is contained in the CuL_4_ equatorial plane. (**d**) The head and tails of the magnetic orbital.

To illustrate how spin exchanges of transition metal magnetic ions are controlled by the ligand p-orbitals in their magnetic orbitals, we consider various spin exchanges involving Cu^2+^ (d^9^, S = 1/2) ions as an example, which typically form axially elongated CuL_6_ octahedra (Figure 6a). The energies of their d-states are split as (xz, yz) < xy < 3z^2^–r^2^ < x^2^–y^2^ (Figure 6b) so that the magnetic orbital of each Cu^2+^ ion is represented by the x^2^–y^2^ state, in which the Cu x^2^–y^2^ orbital induces σ-antibonding with the p-orbitals of four equatorial ligands L (Figure 6c). In this section, we examine how the various types of spin exchanges associated with Cu^2+^ ions are controlled by the ligand p-orbitals of their x^2^–y^2^ states. The major component of the magnetic orbital of a Cu^2+^ ion (Figure 6d) is the Cu d-orbital (i.e., the magnetic orbital “head”), and the minor component the ligand p-orbitals (i.e., the magnetic orbital “tail”). In this section, we probe how the nature and strengths of M–L–M, M–L…L–M and M–L…A…L–M-type exchanges are determined by how the ligand p-orbital tails of their magnetic orbitals are arranged in their exchange paths.

### 4.1. M–L…L–M and M–L…A…L–M Spin Exchanges

**Figure 7:**
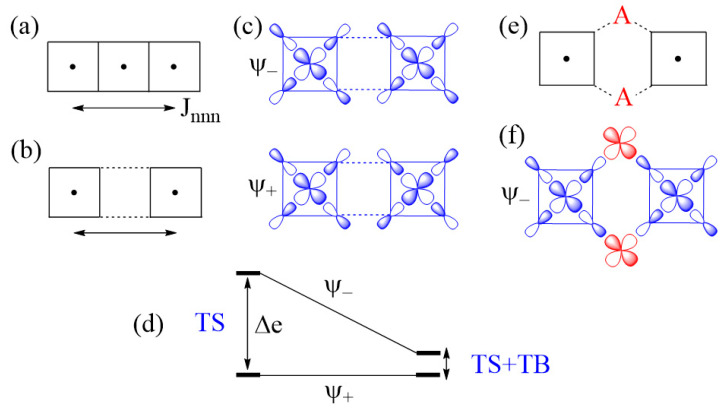
(**a**) The next-nearest-neighbor spin exchange J_nnn_ in a CuL_2_ ribbon chain made up of edge-sharing CuL_4_ square planes. (**b**) A case of strong Cu–L…L–Cu exchange, which represents the next-nearest-neighbor exchange J_nnn_. (**c**) The in-phase and out-of-phase combinations of the two magnetic orbitals, $\Psi_{+}$ and $\Psi_{-}$, respectively, associated with the Cu–L…L–Cu exchange. (**d**) The large energy split Δe resulting from the through-space (TS) interaction in the Cu–L…L–Cu exchange becomes small in the Cu–L…A…L–Cu exchange as a result of the through-bond (TB) interaction that occurs with the $\Psi_{-}$ state. (**e**) A Cu–L…A…L–Cu exchange generated when each L…L contact is bridged by a d^0^ metal cation A. (**f**) The bonding interaction of the $\Psi_{-}$ state with the d_π_ orbital of A.

The next-nearest-neighbor (nnn) spin exchange J_nnn_ (Figure 7a) that occurs in a CuL_2_ (L = O, Cl, Br) ribbon chain, is obtained by sharing the opposite edges of CuL_4_ square planes. This spin exchange is an example of a strong Cu–L…L–Cu exchange (Figure 7b), when the L…L contact distance is in the vicinity of the van der Waals distance. The latter will be assumed to be the case in what follows. The two magnetic orbitals interact across the L…L contacts through the overlap of their p-orbital tails. This through-space interaction leads to the in-phase and out-of-phase combinations ($\Psi_{+}$ and $\Psi_{-}$, respectively) of the magnetic orbitals (Figure 7c), with energy split Δe between the two (Figure 7d). This makes the AFM component J_AF_ non-zero. The overlap electron density associated with the interacting p-orbital tails is nearly zero, so the FM component J_F_ is practically zero. As a result, J_nnn_ becomes AFM.

In the M–L…L–M spin exchange J_nnn_ discussed above, there are two equivalent exchange paths due to the ribbon structure. Suppose that each L…L contact of the M–L…L–M exchange path is bridged by a d^0^ transition metal cation A (e.g., V^5+^ and W^6+^) to form an M–L…A…L–M spin exchange (Figure 7e). We now analyze the relative strengths of the M–L…L–M and M–L…A…L–M spin exchanges. Across the L…L contact of the M–L…L–M path, the p-orbital tails of L are combined in-phase in $\Psi_{+}$, but out-of-phase in $\Psi_{-}$. Thus, the empty d_π_ orbital of the cation A interacts in-phase with $\Psi_{-}$ (Figure 7f) to lower the energy of $\Psi_{-}$, but it does not interact with $\Psi_{+}$, therefore the energy of $\Psi_{+}$ is unaffected. This selective interaction of the bridging d^0^ cation A with the L…L contact of the M–L…L–M spin exchange has a dramatic consequence on the strength of an M–L…A…L–M spin exchange. When the M–L…L–M exchange has a strong through-space interaction, the through-bond interaction reduces the large energy split Δe to a small value, thereby weakening the overall M–L…A…L–M spin exchange (Figure 7d).

**Figure 8:**
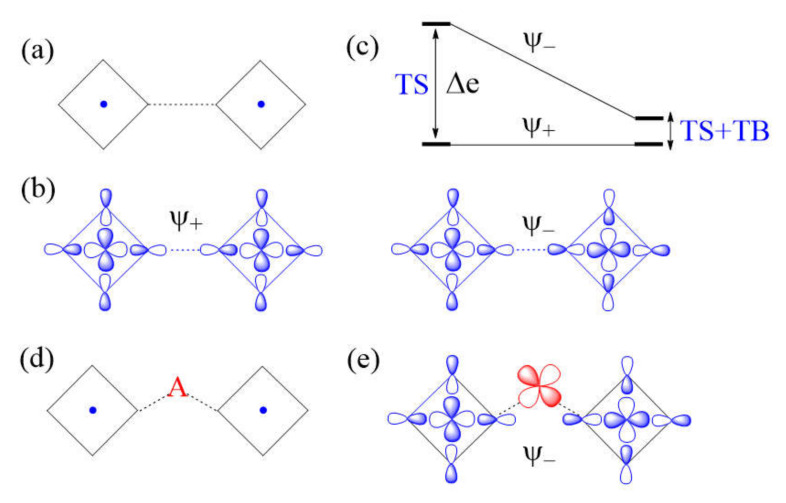
(**a**) A case of strong Cu–L…L–Cu spin exchange where the two Cu–L bonds leading to the L…L contacts are linear. (**b**) The in-phase and out-of-phase combinations ($\Psi_{+}$ and $\Psi_{-}$, respectively) of the two magnetic orbitals, resulting from the through-space (TS) interactions. (**c**) The energy split Δe between $\Psi_{+}$ and $\Psi_{-}$ is large when the overlap between the p-orbital tails is large. (**d**) A Cu–L…A…L–Cu exchange generated when the L…L contact is bridged by a d^0^ metal cation A. (**e**) The bonding interaction of the $\Psi_{-}$ state with the d_π_ orbital of A. (**f**) The large energy split Δe resulting from the through-space (TS) interaction in the Cu–L…L–Cu exchange becomes small in the Cu–L…A…L–Cu exchange as a result of the through-bond (TB) interaction that occurs primarily with the $\Psi_{-}$ state.

Another example of a strong M–L…L–M exchange occurs when the p-orbital tails of L are pointed to each other along the L…L contact (Figure 8a). The through-space interaction between the magnetic orbitals leads to the in-phase and out-of-phase combinations, $\Psi_{+}$ and $\Psi_{-}$, respectively (Figure 8b), with a large energy split Δe between the two (Figure 8c) and a negligible overlap electron density between the interacting p-orbital tails. As a result, the M–L…L–M spin exchange becomes AFM. If the L…L contact of an M–L…L–M exchange path is bridged by a d^0^ transition metal cation A to form an M–L…A…L–M spin exchange (Figure 8d), only the $\Psi_{-}$ state of the M–L…L–M path interacts effectively with one of the empty d_π_ orbitals of A (Figure 8e). Thus, a strong through-space interaction in the M–L…L–M exchange leads to a weak overall M–L…A…L–M spin exchange due to the effect of the through-bond interaction (Figure 8c).

**Figure 9:**
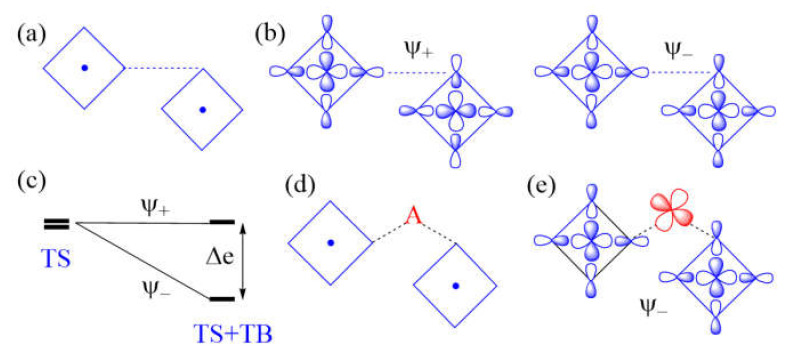
(**a**) A case of weak Cu–L…L–Cu spin exchange, where the two Cu–L bonds leading to the L…L contacts are orthogonal. (**b**) The in-phase and out-of-phase combinations ($\Psi_{+}$ and $\Psi_{-}$, respectively) of the two magnetic orbitals. (**c**) The vanishing energy split Δe resulting from the through-space (TS) interaction in the Cu–L…L–Cu exchange is enhanced in the Cu–L…A…L–Cu exchange as a result of the through-bond (TB) interaction that occurs primarily with the $\Psi_{-}$ state. (**d**) A Cu–L…A…L–Cu exchange generated when the L…L contact is bridged by a d^0^ metal cation A. (**e**) The in-phase interaction of the $\Psi_{-}$ state with the d_π_ orbital of A.

An example of very weak Cu–L…L–Cu exchange is shown in Figure 9a, in which the two magnetic orbitals are arranged such that the p-orbital tails are orthogonal to each other (Figure 9b), and their overlap vanishes so that the energy split between $\Psi_{+}$ and $\Psi_{-}$ vanishes (i.e., Δe = 0) (Figure 9c) so that J_AF_ = 0. In addition, J_F_ should vanish because the overlap electron density resulting from the p-orbital tails will be practically zero. Then, the spin exchange J would be zero. If the L…L contact of such an M–L…L–M exchange path is bridged by a d^0^ transition metal cation A to form an M–L…A…L–M spin exchange (Figure 9d), the $\Psi_{-}$ state of the M–L…L–M path interacts with the empty d_π_ orbital of A, thereby lowering its energy (Figure 9e) while that of the $\Psi_{+}$ state is unchanged. Thus, when the M–L…L–M exchange has a very weak through-space interaction, the through-bond interaction induces the large energy split Δe, so that the overall M–L…A…L–M spin exchange becomes strong (Figure 9f).

In short, a strong M–L…L–M exchange becomes a weak M–L…A…L–M exchange when the L…L linkage is bridged by d^0^ cations, while a weak M–L…L–M exchange becomes a strong M–L…A…L–M exchange when the L…L linkage is bridged by a d^0^ cation.

### 4.2. M–L–M Spin Exchanges

**Figure 10:**
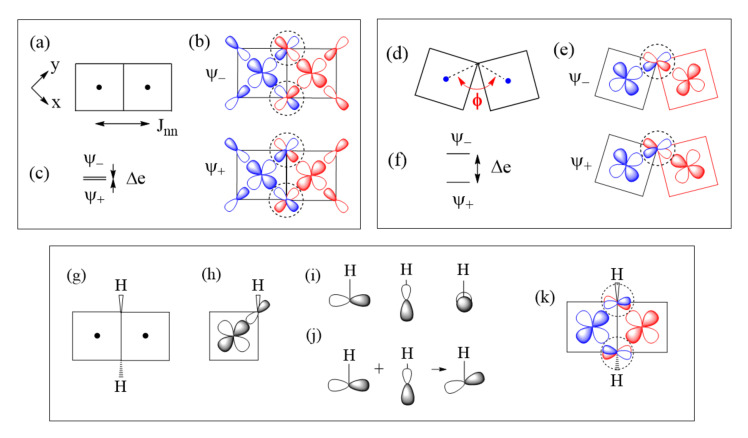
(**a**–**c**) A Cu–L–Cu spin exchange with ∠M–L–M angle ϕ = 90°: This occurs in a Cu_2_L_6_ dimer shown in (**a**), which is made up of two coplanar CuL_4_ square planes by sharing an edge. The in-phase and out-of-phase combinations of the two magnetic orbitals in (**b**), and the energy split Δe between the two in (**c**). (**d**–**f**) A Cu–L–Cu spin exchange with ∠M–L–M angle ϕ > 90°: This occurs when two non-coplanar CuL_4_ square planes are corner-shared as in (**d**). The in-phase and out-of-phase combinations of the two magnetic orbitals in (**e**), and the energy split Δe between the two in (**f**). (**g**–**k**) Effect of the molecular anions OH^−^ on the spin exchange of the edge-sharing Cu_2_O_6_ dimer: the shared edge consists of two OH^−^ ions in (**g**); the p-orbital tail of one OH^−^ ligand in a CuO_4_ square plane expected if the OH^−^ is treated as O^2−^ in (**h**); the three oxygen lone pairs associated with an isolated OH^−^ in (**i**); the tilting of the O 2p-orbital tail by mixing two oxygen lone pairs in (**j**); and the arrangement of the tilted O 2p-tails at the bridging O atoms of the Cu_2_O_6_ dimer in (**k**).

The Goodenough–Kanamori rules cover these types of spin exchanges [5,6,7,8,9]. For the sake of completeness, we discuss these types of spin exchanges from the viewpoint of the ligand p-orbital tails on the basis of Equations (18) and (19). Let us consider a Cu_2_L_6_ dimer resulting from two CuL_4_ square planes obtained by sharing an edge (Figure 10a), where the ligand L can be O, Cl, or Br. The two magnetic orbitals associated with the nearest-neighbor (nn) spin exchange J_nn_, presented in Figure 10b, interact at the bridging ligands L of the M–L–M paths. If the CuL_4_ units have an ideal square planar shape, the ∠M–L–M angle becomes 90° so that the two p-orbital tails at the bridging ligands L are orthogonal to each other. Thus, as discussed in Section 3, the overlap integral between them is zero. Therefore, the in-phase and out-of-phase combinations of the two magnetic orbitals (Figure 10b) is not split in energy, so Δe = 0 (Figure 10c) and J_AF_ = 0. However, the overlap electron density between the two p-orbital tails at the bridging ligand L is not zero, i.e., J_F_ > 0. Thus, the M–L–M spin exchange becomes FM. When the ∠M–L–M angle deviates from 90° (Figure 10d), the two p-orbital tails at the bridging ligands L are no longer orthogonal to each other so the overlap integral between them is non-zero. Therefore, the in-phase and out-of-phase combinations of the two magnetic orbitals (Figure 10e) differ in energy, so Δe > 0 (Figure 10f) and J_AF_ is non-zero. The overlap electron density between the two p-orbital tails is non-zero, so J_F_ is non-zero. Thus, whether the spin exchange is FM or AFM depends on which component, J_F_ or J_AF_, dominates, which in turn depends on the ∠M–L–M angle ϕ. Typically, the angle ϕ where FM changes to AFM is slightly greater than 90° due to the involvement of the ligand s-orbital [15], which is commonly neglected for simplicity.

So far in our discussion, it has been implicitly assumed that each main-group ligand L exists as a spherical anion (e.g., each O as an O^2−^ anion, and each Cl atom as a Cl^−^ anion). However, this picture is not quite accurate when the ligand atom makes a strong covalent bonding with another main-group element to form a molecular anion such as OH^−^. Suppose that each O atom on the shared edge of the CuO_6_ (L = O) dimer is not a O^2−^ but an OH^−^ anion (Figure 10g). Then, the ligand p-orbital tail on that O cannot be the p-orbital pointed along one lobe of the Cu x^2^–y^2^ orbital (Figure 10h) because it is incompatible with the O–H bonding, which has three directional O lone pairs depicted in Figure 10i. To satisfy both the strong covalent-bonding with H and the weak covalent-bonding with Cu, the O lone pair of OH^−^ tilts slightly toward one lobe of the x^2^–y^2^ orbital (Figure 10j). As a result, the ligand p-orbital tails, arising from the two magnetic orbitals at the bridging O atoms, are not orthogonal as in Figure 10b but become more parallel to each other (Figure 10k). As a result, the spin exchange between the two Cu^2+^ ions in Figure 10g becomes AFM (see below). Another molecular anion of interest is the carbonate ion CO_3_^2−^, in which each O atom makes a strong covalent bond with C; therefore, the O atoms of CO_3_^2−^ should not be treated as isolated O^2−^ anions in their coordination with transition metal cations. In general, the presence of molecular anions such as OH^−^ and CO_3_^2−^ in a magnetic solid makes it difficult to deduce, on a qualitative reasoning, what its spin lattice would be. This is where the quantitative energy-mapping analysis is indispensable, because it does not require any qualitative reasoning.

### 4.3. Qualitative Rules for Spin Exchanges Based on the p-Orbital Tails of Magnetic Orbitals

In a magnetic orbital of an ML_n_ polyhedron, the d-orbital of M dictates by its symmetry which p-orbitals of the ligands L become the p-orbital tails. From the viewpoint of orbital interaction, a spin exchange between magnetic ion is none other than the interaction between their magnetic orbitals. The latter is caused by the interaction between their p-orbital tails, not by that between their d-orbital heads. In other words, a spin exchange is not a “head-to-head” interaction but a “tail-to-tail” interaction. By considering these tail-to-tail interactions described above, we arrive at the following four qualitative rules governing the nature and strengths of the M–L…L–M and M–L…A…L–M type exchanges under the assumption that the L…L contact distance is in the vicinity of the van der Waals distance:

(1) When the p-orbital tails generate a large overlap integral but a small overlap electron density, the M–L…L–M exchange is AFM.
(2) When the p-orbital tails generate a small overlap integral but a large overlap electron density, the M–L…L–M exchange is FM.
(3) When the p-orbital tails generate neither a non-zero overlap integral nor a non-zero overlap electron density, the M–L…L–M exchange vanishes.
(4) When the M–L…L–M exchange is strongly AFM, the corresponding M–L…A…L–M becomes a weak exchange. When the M–L…L–M exchange is a weak exchange, the corresponding M–L…A…L–M becomes strongly AFM.

These rules on the M–L…L–M and M–L…A…L–M exchanges should be used together with the Goodenough–Kanamori rules in choosing a proper set of spin exchanges to evaluate using the energy-mapping analysis based on DFT+U or DFT+hybrid calculations. In principle, this analysis can provide quantitative values for any possible exchanges of a given magnetic system. However, even this quantitative tool cannot determine the value of any spin exchange unless it is included in the set of spin exchanges for the energy-mapping analysis. It is paramount to consider in detail the structural features governing the strengths of spin exchanges in order not to miss exchange paths crucial for defining the correct spin lattice of a given solid.

## 5. Representative Examples

In Section 4, we analyzed the structural features governing the nature of the three types of spin exchanges, i.e., M–L–M, M–L…L–M and M–L…A…L–M, which occur in various magnetic solids. This section will discuss the occurrence of these exchanges in actual magnetic solids by analyzing the crystal structures and magnetic properties of four representative magnetic solids, α-CuV_2_O_6_, LiCuVO_4_, (CuCl)LaNb_2_O_7_ and Cu_3_(CO_3_)_3_(OH)_3_. α-CuV_2_O_6_, LiCuVO_4_, and (CuCl)LaNb_2_O_7_ were chosen to show that correct spin lattices can be readily predicted by the qualitative rules of Section 4.3, although they have to be confirmed by performing the energy-mapping analyses. Azurite Cu_3_(CO_3_)_2_(OH)_2_ was chosen to demonstrate that the spin lattice of a certain magnetic system cannot be convincingly deduced solely on the basis of the qualitative rules. The magnetic ions of such a system are coordinated with molecular anions in which the first-coordinate main-group ligands L make strong covalent bonds with other main-group elements (e.g., H in the OH^−^ ion, and C in the CO_3_^2−^ ion). In such a case, use of the energy-mapping analysis is the only recourse with which to find the spin lattice correct for a given system.

### 5.1. Two-Dimensional Behavior of α-CuV_2_O_6_

**Figure 11:**
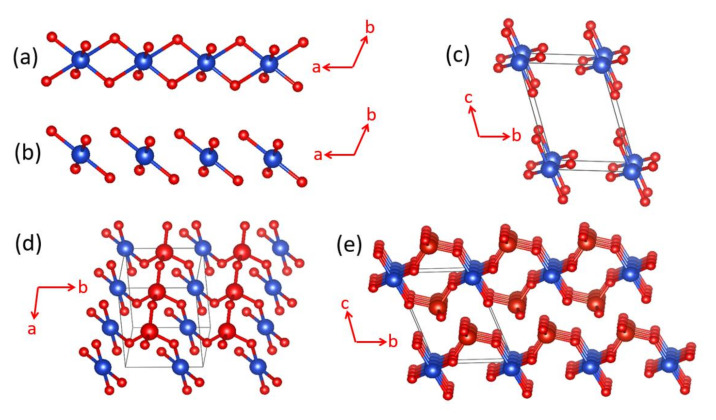
Crystal structure of α-CuV_2_O_6_, where the blue spheres represent the Cu atoms, and the large and small red spheres the V and O atoms, respectively: (**a**) A CuO_4_ chain along the a-direction, which is made up of edge-sharing, axially-elongated, CuO_6_ octahedra. (**b**) A stack of CuO_4_ square planes along the a-direction, which results from the chain of edge-sharing CuO_6_ octahedra by removing the axial Cu-O bonds. (**c**) Stacks of CuO_4_ square planes running along the c-direction. (**d**) One sheet of CuO_4_ stack chains parallel to the a–b plane condensed with chains of corner-sharing VO_4_ tetrahedra on one side of the sheet. (**e**) Stacking of CuV_2_O_6_ layers forming α-CuV_2_O_6_.

**Figure 12:**
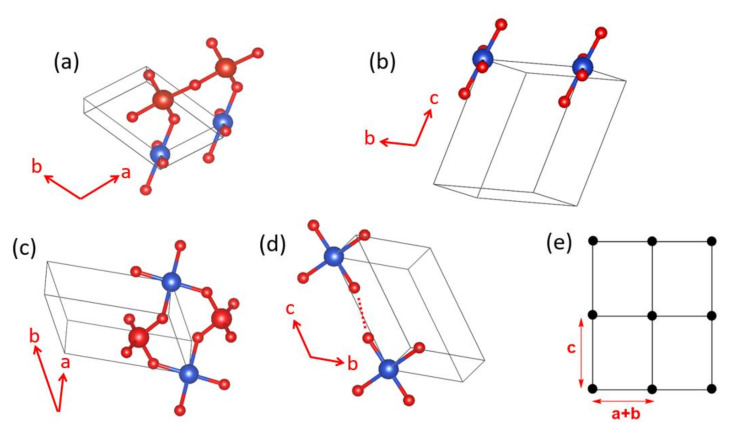
Spin exchange paths and spin lattice of α-CuV_2_O_6_: (**a**) Spin exchange along the b-direction, J_b_; (**b**) Spin exchange along the c-direction, J_c_; (**c**) Spin exchange along the a-direction, J_a_; (**d**) Spin exchange along the (a + b)-direction, J_a+b_; (**e**) Rectangular spin lattice made up of J_c_ and J_a+b_.

The magnetic properties of α-CuV_2_O_6_ were initially analyzed in terms of a one-dimensional (1D) spin S = 1/2 Heisenberg chain model with uniform nearest-neighbor AFM spin exchange [16,17,18]. However, a rather high Néel temperature of ~22.4 K indicated the occurrence of a substantial interchain spin exchange of the order of 50% of the intrachain exchange, casting serious doubts on the applicability of a simple chain description. α-CuV_2_O_6_ consists of CuO_4_ chains, made up of edge-sharing CoO_6_ octahedra, which run along the a-direction (Figure 11a). If the two axially elongated Cu–O bonds are removed from each CuO_6_ octahedron to identify its CuO_4_ equatorial plane containing the magnetic orbital, one finds that each CuO_4_ chain of edge-sharing CoO_6_ octahedra becomes a chain of stacked CuO_4_ square planes (Figure 11b). In each stack-chain along the a-direction, adjacent CuO_4_ square planes are parallel to each other such that the adjacent magnetic orbitals generate neither a non-zero overlap nor a non-zero overlap electron density. The same is true between adjacent CuO_4_ square planes along the b-direction (Figure 11c and Figure 12a). However, between adjacent CuO_4_ square planes along the c-direction, an almost linear Cu–O…O–Cu contact occurs with O…O distance of 2.757 Å (Figure 11c and Figure 12b), slightly shorter than the van der Waals distance of 2.80 Å. Thus, this Cu–O…O–Cu spin exchange along the c-direction (J_c_) should be substantial.

Each sheet of CuO_4_ stack-chains parallel to the a–b plane is corner-shared with V_2_O_6_ chains, above and below the sheet (Figure 11d), to form layers of composition CuV_2_O_6_ (Figure 11e). Each V_2_O_6_ chain is made up of corner-sharing VO_4_ tetrahedra containing V^5+^ (d^0^, S = 0) ions. As a consequence, adjacent CuO_4_ square planes are corner-shared with V_2_O_6_ chains (Figure 12c). The adjacent CuO_4_ square planes are not bridged by a single VO_4_ tetrahedron; therefore, the spin exchange along the a-direction, J_a_, is expected to be weak. Along the (a + b)-direction, two adjacent CuO_4_ square planes are bridged by VO_4_ to form two Cu–O…V^5+^…O–Cu paths, in which the two Cu–O bonds in each path have a near-orthogonal arrangement.

As already discussed (Figure 10), this Cu–O…V^5+^…O–Cu spin exchange should be substantial due to the through-bond effect of the V^5+^ cation. Consequently, the spin lattice of α-CuV_2_O_6_ must be described by a two-dimensional (2D) rectangular lattice defined by J_a+b_ and J_c_. In support of this analysis, the energy-mapping analysis based on DFT+U calculations with U_eff_ = 4 eV show that J_a+b_ and J_c_ are the two dominant spin exchanges, and are nearly equal in magnitude, namely, J_a+b_ = 86.8 K and J_c_/J_a+b_ = 0.88 [19]. In agreement with this finding, one re-investigation of the magnetic properties of α-CuV_2_O_6_ clearly attested a 2D S = 1/2 rectangular spin lattice model with an anisotropy ratio of 0.7 [19]. The magnetic structure determined from neutron powder diffraction data was in best agreement with these DFT+U calculations. In terms of chemical bonding, α-CuV_2_O_6_ consists of CuV_2_O_6_ layers stacked along the c-direction. There is no chemical bonding between adjacent layers; only van der Waals interactions. In terms of magnetic bonding, however, α-CuV_2_O_6_ consists of 2D spin lattices parallel to the (a + b)-c plane. There is negligible magnetic bonding perpendicular to this plane.

### 5.2. One-Dimensional Chain Behavior of LiCuVO_4_

**Figure 13:**
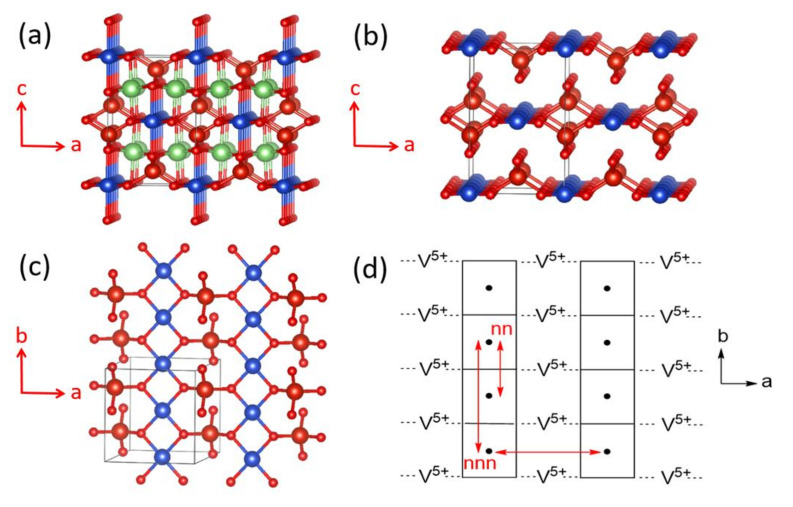
(**a**) The crystal structure of LiCuVO_4_ viewed approximately along the b-direction, where the blue and green spheres represent the Cu and Li atoms, respectively, and the large and small red spheres represent the V and O atoms, respectively. (**b**) The CuVO_4_ lattice resulting from LiCuVO_4_ by removing the axial Cu–O bonds and Li atoms. (**c**) A perspective view of a single CuVO_4_ layer approximately along the c-direction. (**d**) The spin exchange paths present in a single CuVO_4_ layer, where the labels nn, nnn and a represent the spin exchanges J_nn_, J_nnn_ and J_a_, respectively.

LiCuVO_4_ consists of axially elongated CuO_6_ octahedra, which form edge-sharing CuO_4_ chains along the b-direction, which are corner-shared with VO_4_ tetrahedra containing V^5+^ (d^0^, S = 0) ions (Figure 13a). When the axial Cu–O bonds are deleted, one finds CuVO_4_ layers in which the CuO_2_ ribbon chains are corner-shared by VO_4_ tetrahedra (Figure 13b). A perspective view of a single CuVO_4_ layer approximately along the c-direction (Figure 13c) shows that each VO_4_ tetrahedron bridges two neighboring CuO_2_ ribbon chains. Thus, the spin exchanges of interest are the nearest-neighbor exchanges J_nn_ of the Cu–O–Cu type and the next-nearest-neighbor spin exchange J_nnn_ of the Cu–O…O–Cu type within each CuO_2_ ribbon chain as well as the interchain spin exchange J_a_ along the a-direction of the Cu-O…V^5+^…O-Cu type (Figure 13d).

In the absence of the V^5+^ ion, the exchange J_a_ would be similar in strength to J_nnn_. However, the O…V^5+^…O bridges will weaken the strength of J_a_, as discussed in Figure 7. Furthermore, there is no spin exchange path between adjacent CuVO_4_ layers. Consequently, the spin lattice of LiCuVO_4_ is a 1D chain running along the b-direction, as is the 1D ribbon chain. The major cause for the occurrence of the 1D chain character is the Cu–O…V^5+^…O–Cu spin exchange, which nearly vanishes because the effect of the through-space interaction is canceled by that of the through-bond interaction. In agreement with this reasoning, DFT+U calculations with U_eff_ = 4 eV show that J_nnn_ is strongly AFM (i.e., 208.7 K), while J_nn_ and J_a_ are weakly FM (i.e., J_nn_/J_nnn_ = −0.12, and J_a_/J_nnn_ = −0.08) [20].

**Figure 14:**
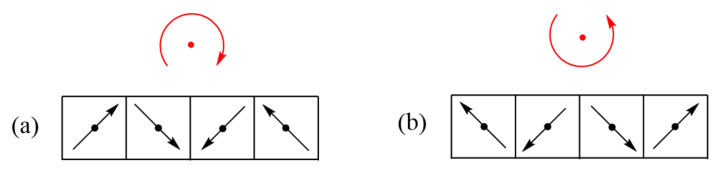
(**a**,**b**) Two cycloids, which are opposite in chirality but are identical in energy.

As in the case of α-CuV_2_O_6_, the magnetic properties of LiCuVO_4_ were initially analyzed in terms of a 1D chain with uniform nearest-neighbor Heisenberg spin exchange [21,22,23]. The magnetic susceptibility of LiCuVO_4_ showed a typical broad maximum at about 28 K, characteristic for 1D behavior with strong intrachain spin exchanges, whereas long-range AFM ordering was detected only below ~2.2 K. The need to modify this simple 1D description was brought about by Gibson et al., who determined the magnetic structure of LiCuVO_4_ from single crystal neutron diffraction [24]. They found that the spins of each CuO_2_ ribbon chain had a cycloid structure (Figure 14a) with adjacent Cu^2+^ moments making an angle of slightly less than 90°. The incommensurate cycloid structure is explained by spin frustration due to competing exchange J_nn_, which is FM, and J_nnn_, which is AFM [20,24,25,26]. If all the spins of a CuO_2_ ribbon chain were to be collinear, the ribbon chain cannot satisfy all nearest-neighbor exchanges FM and all next-nearest-neighbors AFM simultaneously. Thus, the spin arrangement of the CuO_2_ ribbon chain is spin-frustrated. To reduce the extent of this spin frustration, the spins of the ribbon chain adopt a noncollinear spin arrangement. The noncollinear spin arrangement observed for LiCuVO_4_ has a cycloid structure in each ribbon chain, in which the nearest-neighbor spins are nearly orthogonal to each other, while the next-nearest-neighbor spins generate a near-AFM arrangement (Figure 14a) [24,25]. In a cycloid, each successive spin in the CuO_2_ ribbon chain rotates in one direction by a certain angle. Thus, a cycloid structure is chiral in nature, which means that the alternative cycloid structure opposite in chirality but identical in energy is equally probable (Figure 14b) [27].

**Figure 15:**
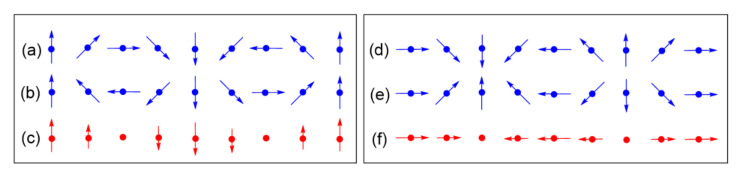
The superposition of the two chiral cycloids in (**a**); (**b**) leads to the transverse SDW in (**c**). The superposition of the two chiral cycloids in (**d**); (**e**) leads to the longitudinal SDW in (**f**). For ease of illustration, the angle of the spin rotation was taken to be 45°.

In general, when the temperature is lowered below a certain temperature, T_SDW_, a moderately spin-frustrated magnetic system gives rise to two cycloids of opposite chirality with equal probability. The resulting superposition of the two (Figure 15a–c) leads to a state known as a spin density wave (SDW) [27,28]. The latter becomes transverse if the preferred spin orientation at each magnetic ion is perpendicular to the SDW propagation direction, but becomes longitudinal if the spin orientation prefers the SDW propagation direction (Figure 15d–f). When the temperature is lowered further below T_SDW_, the electronic structure of the spin-lattice may relax to energetically favor one of the two chiral cycloids so that one can observe a cycloid state at a temperature slightly below T_SDW_. The repeat unit of a cycloid is determined by the spin frustration present in the magnetic system, therefore a cycloid phase is typically incommensurate.

In the cycloid state, LiCuVO_4_ exhibits ferroelectricity [29,30,31], because a cycloid structure lacks inversion symmetry. The polarization of LiCuVO_4_ can be switched with magnetic and electric fields [32,33,34,35]. LiCuVO_4_ has attracted special attention for the possibility of inducing new phases by applying external magnetic fields. Saturation of the Cu moments occurs above ~45 T, depending on the orientation of the crystal [36]. The competition between J_nn_ and J_nnn_ in LiCuVO_4_ is considered a promising setting for unusual bond nematicity and spin nematic phases close to the full magnetic saturation [37,38,39,40,41,42,43,44,45].

### 5.3. Spin Gap Behavior from the CuO_2_Cl_2_ Perovskite Layer of (CuCl)LaNb_2_O_7_

**Figure 16:**
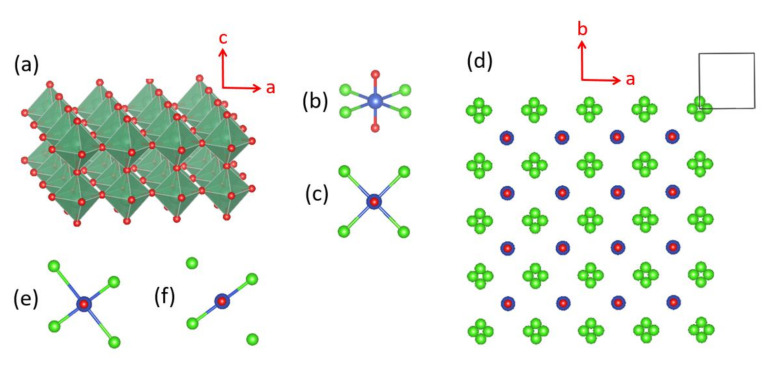
(**a**) One LaNb_2_O_7_ layer of tetragonal (CuCl)LaNb_2_O_7_, where the La atoms at the 12 coordinate sites formed by eight corner-sharing NbO_6_ octahedra are not shown for simplicity. (**b**) Perspective and (**c**) projection views of a CuCl_4_O_2_ octahedron with 4-fold rotational symmetry around the O–Cu–O axis aligned along the c-direction. (**d**) A projection view of a CuClO_2_ layer of tetragonal (CuCl)LaNb_2_O_7_ in which every Cl site is split into four positions. (**e**,**f**) Projection views of the Jahn–Teller distorted CuCl_4_O_2_ octahedron, where the axially elongated Cu–Cl bonds are shown in (**e**), but are not shown in (**f**) for simplicity.

**Figure 17:**
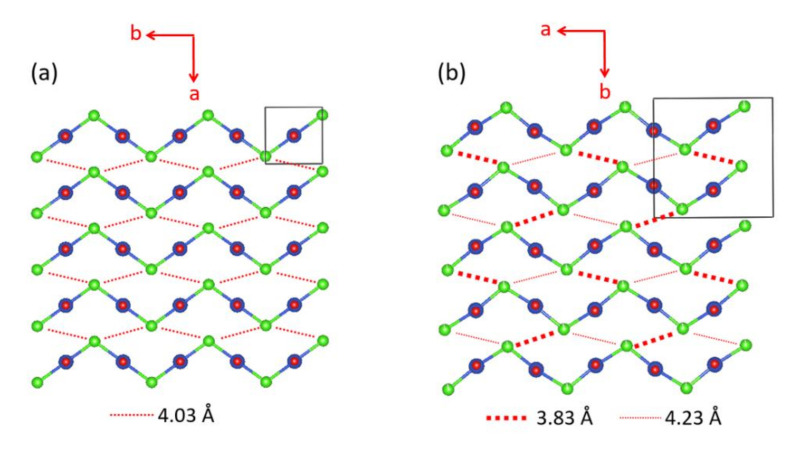
The structures of the CuClO_2_ layer with cooperative Jahn–Teller distortion in (**a**) the tetragonal and (**b**) orthorhombic phases of (CuCl)LaNb_2_O_7_. The Cl–Cu–Cl unit is linear in the tetragonal structure, but is slightly bent in the orthorhombic structure. The latter has an important consequence on the spin exchanges.

(CuCl)LaNb_2_O_7_ consists of CuClO_2_ and LaNb_2_O_7_ layers, which alternate along the c-direction by sharing their O corners [46]. Each LaNb_2_O_7_ layer represents two consecutive layers (Figure 16a) of LaNbO_3_ perovskite. The building blocks of the CuClO_2_ layer are the CuCl_4_O_2_ octahedra with their O atoms at the apical positions forming a linear O–Cu–O bond aligned along the c-direction. Suppose that the CuCl_4_ equatorial planes are square in shape with four-fold rotational symmetry around the O–Cu–O axis (Figure 16b,c), and such CuCl_4_O_2_ octahedra corner-share their Cl atoms to form a perovskite layer CuClO_2_. Then, the highest-occupied d-states of each CuCl_4_O_2_ octahedron are degenerate and have three electrons to accommodate, causing a Jahn–Teller instability of each CuCl_4_O_2_ octahedron. In the CuClO_2_ layer, the CuCl_4_O_2_ octahedra must undergo a cooperative Jahn–Teller distortion. In the tetragonal structure (SG, P4/mmm) of (CuCl)LaNb_2_O_7_ [46], each Cl site is split into four positions (Figure 16d). A Jahn–Teller distortion available to such a CuCl_4_O_2_ octahedron is an axial-elongation, in which one linear Cl–Cu–Cl bond is shortened while lengthening the other linear Cl–Cu–Cl bond, as shown in Figure 16e, which can be simplified as in Figure 16f. By choosing one of the four split positions from each Cl site, it is possible to construct the CuClO_2_ layer with a cooperative Jahn–Teller distortion, as presented in Figure 17a, which shows the Cu–Cl–Cu–Cl zigzag chains running along the b-direction with the plane of each CuCl_2_O_2_ rhombus perpendicular to the a–b plane. Each Cl site has four split positions when all these four possibilities occur equally.

With the local coordinate axes of each CuCl_2_O_2_ rhombus taken as in Figure 17a, then the magnetic orbital of the Cu^2+^ ion can be described as the x^2^–z^2^ state in which Cu x^2^–z^2^ orbital makes σ-antibonding interactions with the p-orbitals of O and Cl. Extended Hückel tight binding calculations [47] for the CuCl_2_O_2_ rhombus show that in the magnetic orbital of the CuCl_2_O_2_ rhombus, the Cu^2+^ ion is described by 0.646 (3z^2^–r^2^) − 0.272 (x^2^–y^2^). The latter is rewritten as:

$$\begin{array}{ll} 0.646(3z^{2}-r^{2})-0.272(x^{2}-y^{2}) & \approx \;[2(3z^{2}-r^{2})-(x^{2}-y^{2})]/3\\ & \propto \;(z^{2}-x^{2})+0.25(x^{2}-y^{2})\;\approx \;(z^{2}-x^{2}) \end{array}$$

Namely, it is dominated by the (z^2^–x^2^) character. This is consistent with the NMR/NQR study of (CuCl)LaNb_2_O_7_, which showed that the d-state of the Cu^2+^ ion is mostly characterized by 3z^2^–r^2^ with some contribution of x^2^–y^2^ [48]. 

**Figure 18:**
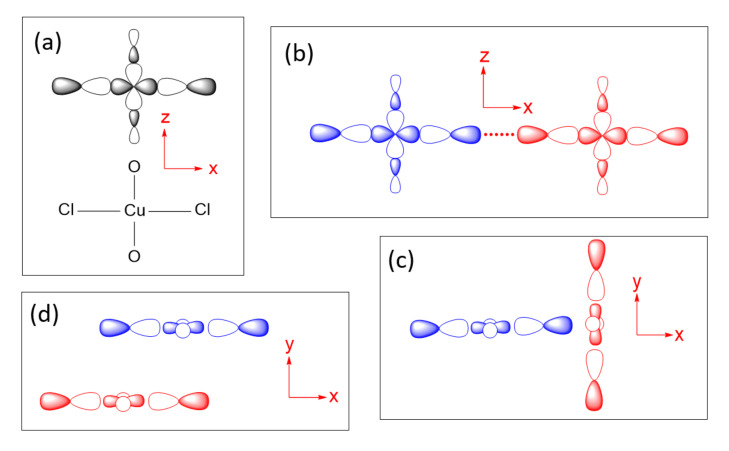
(**a**) The magnetic orbital lying in the CuCl_2_O_2_ rhombus plane, which is best described as the x^2^–z^2^ state. (**b**–**d**) Three different types of the magnetic orbital arrangements found in the CuClO_2_ layers of orthorhombic CuCl(LaV_2_O_7_). In this idealized representation of the CuCl_2_O_2_ rhombus, the slight bending of the Cl–Cu–Cl linkage is neglected for clarity.

We note that the CuCl_2_O_2_ rhombuses of each Cu–Cl–Cu–Cl zigzag chain make Cu–Cl…Cl–Cu contacts of Cl…Cl = 4.03 Å with its adjacent zigzag chains (Figure 17a). The Cl p-orbitals of the (z^2^–x^2^) magnetic orbital (Figure 18a) are pointed approximately along the Cl…Cl contact, so their overlap is substantial. In all other spin exchange paths, the Cl p-orbitals in their magnetic orbitals are not arranged to overlap well. Thus, only the exchange along the Cu–Cl…Cl–Cu direction is expected to be substantially AFM. Then the spin lattice of the CuClO_2_ layer would be 1D Heisenberg uniform AFM chains running along the Cu–Cl…Cl–Cu directions, e.g., the (a + 2b)- and (a − 2b)-directions (Figure 17a). Uniform AFM chains do not have a spin gap, but the magnetic properties of (CuCl)LaNb_2_O_7_ reveal a spin gap behavior [49]. Thus, the tetragonal structure of (CuCl)LaNb_2_O_7_ is inconsistent with experiment. If one discards the possibility of cooperative Jahn-Teller distortions, one can generate several nonuniform clusters made up of distorted CuCl_4_O_2_ octahedra [50]. However, the latter would lead to several different spin gaps rather than that observed by Kageyama et al. [49], who showed that the magnetic susceptibility of (CuCl)LaNb_2_O_7_ can be approximated by an isolated spin dimer model with the intradimer distance of approximately 8.8 Å, which corresponds to the fourth-nearest-neighbor Cu…Cu distance. The spin-gap behavior of (CuCl)LaNb_2_O_7_ was surprising, given the belief of the square lattice arrangement of Cu^2+^ ions is spin-frustrated [49,51,52]. This led to several DFT studies designed to find the precise crystal structure of (CuCl)LaNb_2_O_7_ [53,54,55], leading to the conclusion that an orthorhombic structure of space group Pbam is correct for (CuCl)LaNb_2_O_7_ [54,55].

The cause for the spin gap behavior of (CuCl)LaNb_2_O_7_ is found from its orthorhombic structure (SG, *Pbam*) (Figure 17b) [56,57], in which the arrangement of the Cu^2+^ ions are no longer tetragonal so that adjacent Cu–Cl–Cu–Cl zigzag chains have two kinds of Cl…Cl contacts (i.e., 3.83 and 4.23 Å), and the Cu–Cl…Cl–Cu chains become alternating with shorter and longer Cl…Cl contacts. Furthermore, although the Cl–Cu–Cl unit of each CuCl_2_O_2_ rhombus is slightly bent in the orthorhombic structure, the latter has an important consequence on the spin exchanges (see below).

**Figure 19:**
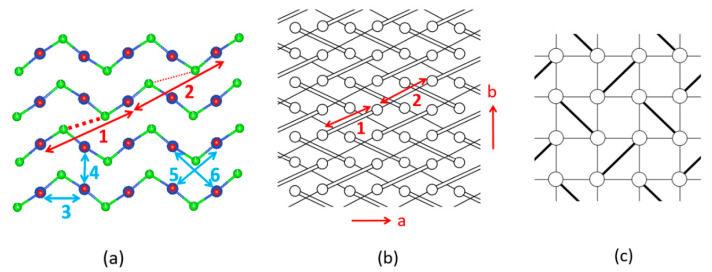
(**a**) Six spin exchange paths of the CuClO_2_ layer in orthorhombic CuCl(LaV_2_O_7_), and the numbers 1–6 refer to J_1_–J_6_, respectively. (**b**) The J_1_–J_2_ AFM alternating chains the CuClO_2_ layer. Only the Cu^2+^ ions are shown as empty circles, with J_1_ and J_2_ paths represented by cylinders and lines, respectively. (**c**) The simplified spin lattice of the CuClO_2_ layer generated by the three strongest spin exchanges, which is the Shastry–Sutherland spin lattice.

The six spin exchange paths J_1_–J_6_ of the CuClO_2_ layer are depicted in Figure 19a. In the spin exchanges J_1_ and J_2_, the Cl p-orbital tails are approximately pointed toward each other (Figure 18b). J_3_ is a Cu–Cl–Cu exchange with ∠Cu–Cl–Cu angle somewhat greater than 90° (namely, 109.0°). In J_4_ and J_5_, the Cl p-orbital tails are approximately orthogonal to each other (Figure 18c), but they differ due to the bending in the Cl–Cu–Cl units. In the J_6_ path, the Cl p-orbital tails are not pointed toward to each other but are approximately parallel to each other (Figure 18d). In the exchange paths J_1_ and J_2_, the Cu–Cl…Cl linkage is more linear and the Cl…Cl contact is shorter in J_1_. This suggests that J_1_ is more strongly AFM than J_2_, thus the spin lattice of the CuClO_2_ layer is an alternating AFM chain. In agreement with this argument, the energy-mapping analysis based on DFT+U calculations shows that J_1_ = 87.5 K and J_2_/J_1_ = 0.18. In addition, this analysis reveals that J_3_–J_6_ are all FM with J_3_/J_1_ = −0.39, J_4_/J_1_ = −0.38, J_5_/J_1_ = −0.14 and J_5_/J_1_ = −0.04 [56]. It is of interest to note that the strongest AFM exchange J_1_ is the fourth-nearest-neighbor spin exchange, with a Cu…Cu distance of 8.53 Å [49]. As shown in Figure 19b, the spins of the CuClO_2_ layer form alternating AFM chains. Chemically, the Cu–Cl zigzag chains run along the a-direction. In terms of magnetic bonding, however, the spins of the CuClO_2_ layer consist of J_1_-J_2_ alternating AFM chains not only along the (a + 2b)-direction but also along the (−a + 2b)-direction. This explains why the magnetic susceptibility of (CuCl)LaNb_2_O_7_ exhibits a spin gap behavior. Due to the bending of the Cl–Cu–Cl units, the Cl p-orbital tail of one CuCl_2_O_2_ rhombus is pointed toward one Cl atom (away from both Cl atoms) of the other rhombus in the J_4_ (J_5_) path. This makes J_4_ more strongly FM than J_5_ is. J_3_ is strongly FM despite the fact that the ∠Cu–Cl–Cu angle is somewhat greater than 90°, probably because the Cl 3p orbital tails are more diffuse than the 2p-orbital tails of the second-row ligand (e.g., O). If the spin lattice of the CuClO_2_ layer is described by using the three strongest spin exchanges, namely, the AFM exchange J_1_ as well as the FM exchanges J_3_ and J_4_, then the resulting spin lattice is topologically equivalent to the Shastry–Sutherland spin lattice (Figure 19c) [56,58].

### 5.4. Two Dimensional Magnetic Character of Azurite Cu_3_(CO_3_)_2_(OH)_2_

**Figure 20:**
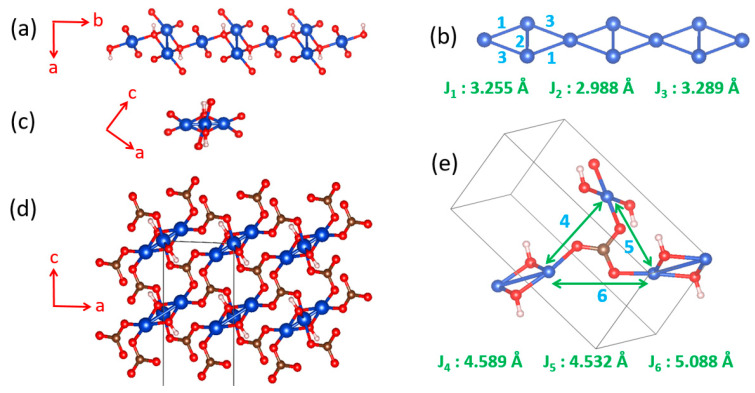
(**a**) The diamond chain made up of CuO_4_ square planar units. (**b**) Definition of the three spin exchanges J_1_–J_3_ in a diamond chain. (**c**) A projection view of the diamond chain along the chain direction. (**d**) The structure of Azurite viewed along the diamond chain direction. (**e**) Definition of the three spin exchanges J_4_–J_6_ between adjacent diamond chains.

Early interests in the magnetic properties of the mineral Azurite, Cu_3_(CO_3_)_2_(OH)_2_, in which Cu^2+^ ions are coordinated with molecular anions CO_3_^2−^ and OH^−^, focused mainly on the paramagnetic AFM ordering transition of the Cu^2+^ moments that occurs at about 1.86 K [59,60,61,62,63]. Renewed interests in the properties of Cu_3_(CO_3_)_2_(OH)_2_ arose from low-temperature high-field magnetization measurements by Kikuchi et al. [64], who detected a magnetization plateau extending over a wide field interval between 16 and 26 T or 11 and 30 T, depending on the crystal orientation. Only one-third of the Cu magnetic moments saturate in these field ranges whereas complete saturation of all Cu moments occurs above 32.5 T [64]. In Cu_3_(CO_3_)_2_(OH)_2_, the Cu^2+^ ions form CuO_4_ square planar units with the CO_3_^2−^ and OH^−^ ions. In each CuO_4_ unit, two O atoms come from two CO_3_^2−^ ions, and the remaining two O atoms from two OH^−^ ions. These CuO_4_ units form Cu_2_O_6_ edge-sharing dimers, which alternate with CuO_4_ monomers by corner-sharing to make a diamond chain (Figure 20a,b). Guided by the crystal structure, Kikuchi et al. [64] explained their results on Cu_3_(CO_3_)_2_(OH)_2_ by considering spin frustration in the diamond chains (Figure 20b), to conclude that all spin exchange constants (J_1_, J_2_ and J_3_) in the diamond chains are AFM with J_2_ being the dominant exchange, and that the moment of the Cu^2+^ ion of the monomer is susceptible to external magnetic fields because its spin exchanges with the two adjacent dimers (i.e., 2J_1_ + 2J_3_) are nearly canceled. In questioning this scenario, Gu et al. and Rule et al. suggested that one of the monomer–dimer spin exchanges is FM, implying the absence of spin frustration [65,66,67]. The diamond chain picture had to be revised when the spin exchanges of Cu_3_(CO_3_)_2_(OH)_2_, evaluated using the energy-mapping analysis [68], showed that, although Kikuchi et al.’s description of the diamond chain was correct, Cu_3_(CO_3_)_2_(OH)_2_ is a 2D spin lattice made up of inter-linked diamond chains.

**Figure 21:**
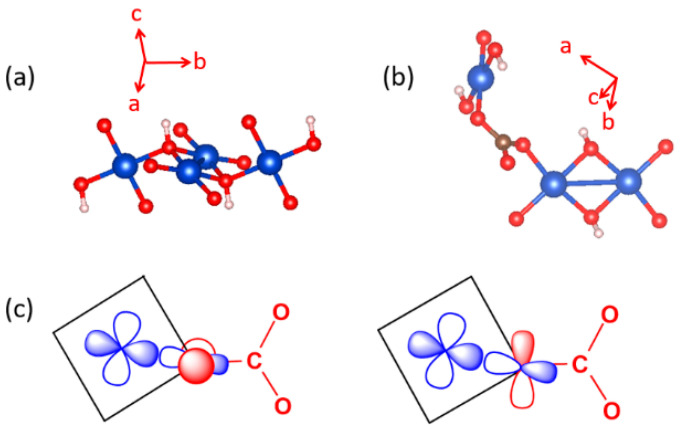
(**a**) A diamond unit of Azurite. (**b**) Arrangement of the CuO_4_ monomer and Cu_2_O_6_ dimer associated with the spin exchange path J_4_. (**c**) Interaction of the O p_π_ and O p_σ_ orbitals of the CO_3_^2−^ ion with the p-orbital tail of the Cu^2+^ ion magnetic orbital.

**Figure 22:**
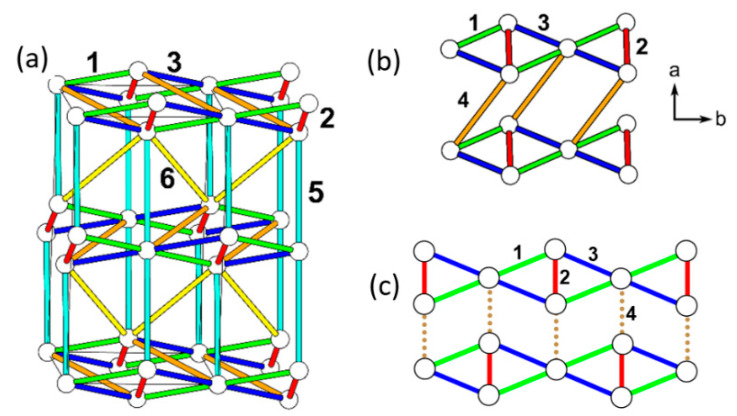
(**a**) Arrangement of the spin exchange paths J_1_–J_6_ in Azurite. (**b**) Arrangement of the four strongest spin exchanges J_1_–J_4_ in Azurite. (**c**) 2D spin lattice describing Azurite.

In the three-dimensional (3D) structure of Cu_3_(CO_3_)_2_(OH)_2_, the diamond chains are interconnected by the CO_3_^2−^ ions. Using the projection view of the diamond chain along the chain direction (Figure 20c), the 3D structure of Cu_3_(CO_3_)_2_(OH)_2_ can be represented as in Figure 20d, which shows that each CO_3_^2−^ ion bridges three different diamond chains. The spin exchange paths of interest for Cu_3_(CO_3_)_2_(OH)_2_ are J_1_–J_3_ in each diamond chain (Figure 20b), as well as J_4_–J_6_ between diamond chains (Figure 20e). In the diamond unit of Azurite (Figure 21a), the two bridging O atoms of the edge-sharing dimer Cu_2_O_6_ form O–H bonds. Thus, the spin exchange J_2_ (Figure 20b) between the two Cu^2+^ ions in the edge-sharing dimer Cu_2_O_6_ are expected to be strongly AFM, as discussed in Section 4.2. The spin exchanges J_1_ and J_3_ of the diamond (Figure 20b) would be similar in strength because their two Cu–O–Cu exchange paths are nearly equivalent due to the near perpendicular arrangement the CuO_4_ monomer plane to the Cu_2_O_6_ dimer plane (Figure 21a). Due to the perpendicular arrangement of the two planes, J_1_ and J_3_ are expected to be weakly AFM and smaller than J_2_. What is difficult to predict without quantitative calculations is the relative strengths of the inter-chain exchanges J_4_–J_6_ (Figure 20e). For example, we consider the J_4_ exchange path shown in Figure 21b. The O p_π_ and O p_σ_ orbitals of the CO_3_^2−^ ion interact with the p-orbital tail of the Cu^2+^ ion magnetic orbital (Figure 21c), which depend not only on the ∠C–O–Cu bond angle, but also on the dihedral angles associated with the spin exchange path (e.g., ∠O–C–O–Cu dihedral angle). It is necessary to resort to the energy-mapping analysis based on DFT+U calculations to find the relative strengths of the spin exchanges J_1_–J_6_. The arrangement of these spin exchange paths in Azurite is presented in Figure 22a. Results of our analysis for J_1_–J_4_ are summarized in Table 1.

Our energy-mapping analysis shows that J_5_ and J_6_ are negligibly weak compared with J_1_–J_4_. The latter four exchanges are all AFM; J_2_ is the strongest while J_1_, J_3_ and J_4_ are comparable in magnitude, which is in support of Kikuchi et al.’s deduction of the spin exchanges J_1_–J_3_ for the diamond chain. The values of J_1_–J_4_ are smaller from the DFT+U calculations with U_eff_ = 5 eV than from those with U_eff_ = 4 eV. This is understandable because the J_AF_ component decreases with increasing the on-site repulsion U (Equation (19)). The strengths of the exchanges J_1_–J_4_ decrease in the order, J_2_ >> J_3_ ≈ J_1_ > J_4_. Thus, in the spin lattice of Azurite, the diamond chains defined by the intrachain exchanges J_2_, J_3_ and J_1_ interact by the interchain exchange J_4_ (Figure 22b). Therefore, the spin lattice of Azurite is a 2D spin lattice described by the exchanges J_1_–J_4_ depicted in Figure 22c. J_3_ and J_1_ are practically equal in strength; therefore, use of a symmetrical diamond chain (i.e., with the approximation J_3_ ≈ J_1_) would be a good approximation. In any event, it is crucial not to neglect the interchain exchange J_4_ because it is comparable in magnitude to J_3_ and J_1_. Alternatively, the spin lattice can be described in terms of the alternating AFM chains defined by J_2_ and J_4_. The 2D spin lattice consists of these alternating AFM chains that are spin-frustrated by the interchain exchanges J_1_ and J_3_.

The importance of interchain exchange indicating that the correct spin lattice of Azurite is not a diamond chain but a 2D net in which the diamond chains are interconnected by the spin exchange spin J_4_ was recognized by Kang et al. in 2009 [68]. This finding, though controversial and vigorously disputed in the beginning, is now accepted as a prerequisite for correctly describing Azurite [69]. The presence of an interchain exchange naturally allows one to understand a long-range AFM order and explain gapped modes in the spin dynamics along the diamond chains [70], and the magnetic contribution to the thermal conductivity [71].

## 6. Concluding Remarks

In this review, we discussed the theoretical foundations of the concept of spin exchanges and analyzed which electronic factors affect their signs and strengths. Noting that a spin exchange between two magnetic ions is mediated by the ligand p-orbital tails, we derived several qualitative rules for predicting whether a given M–L…L–M or M–L…A…L–M exchange would be AFM or FM by inspecting the arrangement of their ligand p-orbital tails in the exchange paths. As long as the L…L distance is in the vicinity of the van der Waals distance, the M–L…L–M or M–L…A…L–M spin exchange can be strong and often stronger than the M–L–M exchanges. In searching for the spin lattice relevant for a given magnetic solid, therefore, it is crucial not to omit the M–L…L–M and M–L…A…L–M spin exchanges when present. The qualitative rules on the M–L…L–M and M–L…A…L–M exchanges, described in Section 4.3, can be used together with the Goodenough–Kanamori rules on M–L–M spin exchanges in selecting a proper set of spin exchanges to evaluate using the energy-mapping analysis.

The important aspect emerging from our discussions is that the nature of a spin exchange is determined by the interactions between the magnetic orbitals. These are governed by the ligand p-orbitals, not by the metal d-orbitals. The essential role that the metal d-orbitals play in any spin exchange is rather indirect. In a magnetic orbital of an ML_n_ polyhedron, the metal d-orbital selects with which ligand p-orbitals it combines and hence determines the nature of the p-orbital tails in the magnetic orbital. The spin exchange between magnetic ions, namely, the interaction between their magnetic orbitals, rests upon the interaction between their p-orbital tails.

## Figures and Tables

**Table 1:** Values of the spin exchanges J_1_–J_6_ determined by DFT+U calculations with U_eff_ = 4 and 5 eV.

	U_eff_ = 4	U_eff_ = 5
J_2_	241.5 K	194.6 K
J_3_/J_2_	0.28	0.27
J_1_/J_2_	0.21	0.19
J_4_/J_2_	0.17	0.16
J_5_/J_2_	−0.01	−0.02
J_6_/J_2_	0.04	0.03
